# Association between self-reported oral health and life satisfaction among China's migrant elderly following children: The mediating effect of social support

**DOI:** 10.3389/fpubh.2023.950942

**Published:** 2023-02-14

**Authors:** Jieru Wang, Mingli Pang, Fanlei Kong

**Affiliations:** ^1^Centre for Health Management and Policy Research, School of Public Health, Cheeloo College of Medicine, Shandong University, Jinan, China; ^2^NHC Key Lab of Health Economics and Policy Research, Shandong University, Jinan, China

**Keywords:** social support, life satisfaction, migrant elderly following children, mediating effect, self-reported oral health

## Abstract

**Purpose:**

Focusing on the life satisfaction of the migrant elderly following children (MEFC) is of great theoretical and practical significance. We aimed to examine the effect of self-reported oral health on life satisfaction among the MEFC in Weifang, China, and to further explore the mediating role of social support on the relationship between self-reported oral health and life satisfaction.

**Methods:**

We conducted a cross-sectional survey for 613 participants using multi-stage random sampling in Weifang, China, in August 2021. The Social Support Rating Scale was used to assess social support for the MEFC. We used the Chinese version of the Geriatric Oral Health Assessment Index (GOHAI) to evaluate self-reported oral health. We assessed life satisfaction for the MEFC via the Satisfaction with Life Scale. The data were scrutinized through descriptive analysis, a chi-square test, a *t*-test, Pearson correlation analysis, and structural equation modeling (SEM).

**Results:**

The mean GOHAI, social support, and life satisfaction scores were 54.95 ± 6.649, 38.89 ± 6.629, and 27.87 ± 5.584, respectively. SEM analysis indicated that the self-reported oral health of the MEFC exerts a positive effect on life satisfaction and social support, and social support has a positive and direct effect on life satisfaction. Social support partially mediates the association between self-reported oral health and life satisfaction (95% confidence interval: 0.023–0.107, *P* < 0.001), with its mediating effect accounting for 27.86% of the total effect.

**Conclusion:**

The mean score of life satisfaction was 27.87 ± 5.584 among the MEFC in Weifang, China, suggesting relatively high life satisfaction. Our findings underscore an empirical association between self-reported oral health and life satisfaction and imply that social support mediates this relationship.

## 1. Introduction

As the largest developing country globally, China has experienced rapid economic and social growth since the reform and opening-up policy (1). With a declining fertility rate and increasing life expectancy, the aging of China's population has become an irreversible trend; China currently has the largest population of older adults worldwide (2). By the end of 2021, the country was home to 263.76 million people aged 60 and older, accounting for 18.9% of the population. Among them, 20.56 million individuals, that is 14.2%, were 65 and older (3). However, with accelerated urbanization in China, a large number of young and middle-aged rural laborers have been migrating to cities to obtain a higher standard of living. In this process, due to improved economic levels and the need for family development, migrants have been increasingly migrating with their family members as a unit (4, 5), and the proportion of older adults among them has been expanding (6). According to the latest data from the National Health Commission, China's older adult migrant population was approximately 18 million in 2015 (7). One possible reason for this phenomenon is that some older adults migrate to cities following their children to take care of their grandchildren. Existing studies refer to older adults aged 60 or above who have left their hometowns and migrated with their children as the migrant elderly following children (MEFC) (8–10).

Life satisfaction is the recognition of, or a positive attitude toward, life as a whole and is an important component of subjective well-being (11). Nearly half of all older Chinese adults were dissatisfied with their lives (12). Another study has indicated that life satisfaction among older Chinese adults needs improvement (13). A study of older adults in East London found that immigrants faced ill-defined social roles, financial constraints, and declining health in their place of immigration compared to native residents, leading to lower life satisfaction and deeper levels of depression (14). For rural older adult migrants who live with their children for reasons such as caring for grandchildren, their material conditions have improved in the city, but the unfamiliar environment has brought more discomfort and emotional despondency (15). In addition, some studies in China have confirmed that it is more difficult for the MEFC to integrate into society due to language barriers in the inflow area, which leads to a decrease in life satisfaction as well as other problems (16, 17). Thus, paying attention to life satisfaction among the MEFC is of great theoretical and practical significance.

The World Health Organization (WHO) defines oral health as a state that is free of oral and facial pain, oral diseases, and disorders that limit an individual's psychosocial wellbeing as well as their ability to bite, chew, smile, and speak (18). A previous study in the United States (US) has shown that oral health was worse among minority or low income older adults (19). Studies in Latin America and the Caribbean have found a high prevalence of dental caries in older adults (20). Another study has also pointed out that the burden of oral disease among African-American and Latino seniors in Northern Manhattan is high (21). These studies indicate that older adults have low oral health and require better oral healthcare services. The Fourth Chinese Oral Health Epidemiological Survey Report demonstrates that the prevalence of dental caries in older adults' permanent teeth is fairly high, implying serious oral health conditions (22). A study on the oral health status of Chinese mainland individuals found that oral health deteriorates with age (23). As one component of overall health, oral health is critical to both general health and wellbeing and greatly affects life satisfaction (24). By negatively affecting physical, psychological, and social aspects, further development of oral diseases could reduce the ability to perform activities of daily living (ADLs), thereby influencing quality of life and life satisfaction (25). Although an association between oral health and life satisfaction has been demonstrated, the underlying mechanisms remain unclear.

Social support refers to the material and moral help provided by various parties in society, including family, relatives, friends, colleagues, organizations, trade unions, and others (26). “Social support” is a general term denoting the diverse types of support that arise from outside the individual and represents the social conduct that accompanies the existence of a vulnerable group. Social support not only provides the resources necessary for older adults to cope with challenges but also has a tremendously positive impact on their physical and mental health, making it the most critical source of wellbeing for older adults (27–29). However, social support in the migrant population is somewhat low according to past research, especially among the MEFC (30, 31). One possible reason is language barriers (32, 33), which may limit social networks and affect social support.

Social support is associated with health (10, 28). Inadequate social support has been associated with poor health outcomes and illnesses such as depression (34). The lack of social support has also been associated with poor dental health, such as low dental function and anterior open bite (35). The results of a cross-sectional study of Swedish residents show that the absence of social support is a factor associated with the reported avoidance of dental care (36). The association between social support and oral health in older adults has been clarified (37) and, specifically, impaired oral health in older adults is positively tied to low social support (38).

Improving social support helps promote life satisfaction among older adults. Song and Fan found that community residents' social support is significantly and positively related to life satisfaction (39). Xiang et al. indicated that urban older adults receive more social support than rural older adults; formal social support has a positive effect on the life satisfaction of older adults, and the moral support provided by their children contributes significantly to their physical health and life satisfaction (40). This is consistent with the results of another study, which found that informal social support, such as that from family members, has a greater impact on rural older adults (41). Although many studies have focused on the social support and life satisfaction of older adults in China, few have used the MEFC as participants. Hence, we hypothesized that oral health would indirectly affect life satisfaction among the MEFC and that the relationship would be mediated by social support.

**Figure 1:**
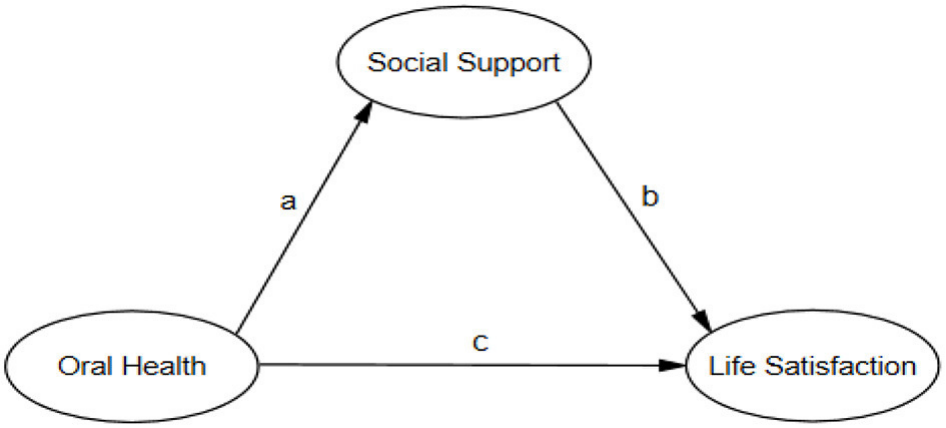
The conceptual framework of mediation model. a refers to the direct path from oral health to social support; b refers to the direct path from social support to life satisfaction; c refers to the direct path from oral health to life satisfaction.

Although many previous studies have explored the relationship between social support and life satisfaction, few have examined the association between self-reported oral health and life satisfaction or between self-reported oral health and social support. Moreover, no study has clarified the link between self-reported oral health and life satisfaction among the MEFC or mentioned the mediating effect of social support on this relationship. Thus, this study aims to investigate the effect of self-reported oral health on life satisfaction and clarify the mediating role of social support between self-reported oral health and life satisfaction among the MEFC in Weifang City, China. The conceptual framework of the mediation model is illustrated in Figure 1.

## 2. Materials and methods

### 2.1. Data and sample

A cross-sectional survey was conducted in Weifang City, Shandong Province, China, in August 2021. Weifang City has four districts, two counties, and six county-level cities, with a total area of 16,167.23 square kilometers (42). As of 2021, the city has a gross domestic product (GDP) of 701.06 billion yuan, with a resident population of 9.4 million and an urbanization rate of 65.2%, which was 0.8 percentage points higher than that in the previous year; the household population was 9.203 million (43). A multi-stage cluster random sampling method was used to select participants. Considering the influence of economic development and geographical location, four urban areas were selected as primary sampling units in the first stage from among 12 subordinate districts in Weifang. In the second stage, four sub-districts were randomly selected from among these four primary sampling units as subsampling units. In the third stage, three communities were selected from each subsampling unit. Finally, in these communities, all migrant older adults aged 60 and above who had migrated to Weifang with their children constituted the total sample. Those who were (1) 60 years old and above; (2) non-local residents of the household registration area; and (3) able to understand Mandarin and communicate effectively with the surveyors were included as participants. This study's enumerators comprised 25 university and master's-level students. Prior to the survey, these enumerators received training in the following areas: research background, questionnaire content, and social survey techniques. The enumerators obtained the respondents' consent before conducting the survey. These enumerators then collected data by conducting face-to-face interviews with the survey respondents lasting approximately 30 min. The questionnaire return rate of the study was therefore 100%. Ultimately, a total of 613 participants were enrolled in the study.

### 2.2. Measurement

An interviewer-administered questionnaire was used to collect data, which included demographic characteristics and three scales measuring social support, self-reported oral health, and life satisfaction.

#### 2.2.1. Demographic traits

The section on sociodemographic traits included the following: *hukou* (rural, urban); gender; age; marital status (married, no spouse); education level (primary school and below, junior high school, high school and above); type of migrant household (from a rural or urban area); migration time (< 4.5 years, 4.5 years≥); and migration reason (taking care of grandchildren, not taking care of grandchildren).

#### 2.2.2. Social support

We used the Social Support Rating Scale (SSRS) to measure social support for the MEFC. The SSRS has been widely used in China and has good reliability and validity (9, 44, 45); it has 10 items, including the following three dimensions: (1) objective support (three items); (2) subjective support (four items); and (3) the utilization of social support (three items). A higher total score indicates that the participant received more social support. The scale has a range of 12–66: (1) a total score of ≤ 22 indicates a low level of social support; (2) a score of 23 ≤ total score ≤ 44 implies a moderate level of social support; and (3) a score of 45 ≤ total score ≤ 66 denotes a high level of social support.

#### 2.2.3. Self-reported oral health

We used the Chinese version of the Geriatric Oral Health Assessment Index (GOHAI) gauge primarily to assess the self-reported oral health of older adults. The GOHAI is now widely used in China as well as abroad. The Chinese version of the GOHAI is divided into three sub-dimensions and 12 items designed to assess different aspects of oral health: (1) physical functioning (four items); (2) psychosocial functioning (five items); and (3) pain or discomfort (three items). GOHAI scores are categorized as follows: 50 and below are defined as low self-reported oral health; 51–56 as moderate self-reported oral health; and 57–60 as high self-reported oral health. In this study, the GOHAI scores also had good reliability and validity (46).

#### 2.2.4. Life satisfaction

We measured life satisfaction among the MEFC using the Satisfaction with Life Scale (SWLS). The SWLS consists of five questions, each of which has seven levels of judgment, from “not at all” to “completely.” Answers are rated on a scale of 1 to 7, respectively. The number of each response is the score for that item; hence, the total score ranges from 5 to 35, with higher scores suggesting greater satisfaction with life. Previous studies have shown that the SWLS has good reliability and validity (47, 48). The participants answered the questions by selecting one option for each sentence according to the degree to which it matched their actual situation.

### 2.3. Statistical analysis

Data analysis was performed using SPSS version 22.0 (SPSS Inc., Chicago, IL, USA) and SPSS Amos 22.0 (IBM Corp., Armonk, NY, USA). A *P*-value ≤ 0.05 was deemed to be statistically significant. First, the descriptive statistics of the subjects' sociodemographic characteristics were obtained. Urban–rural differences in sociodemographic characteristics, self-reported oral health, social support, and life satisfaction were determined using chi-square and *t*-tests. Second, Pearson's correlation analysis was applied to explore the relationship between the sub-scales of self-reported oral health, social support, and life satisfaction. Third, a hypothesized structural equation model was formulated. Maximum likelihood estimation was conducted to evaluate the hypothesized model's fit. Finally, a bootstrap analysis test (in which the sampling process was repeated 1,000 times) was employed to examine the total, indirect, and direct effects (49). The indirect effect (the mediating effect in the study) was regarded as statistically significant if the 95% confidence interval (CI) excluded zero. The structural equation modeling (SEM) process involved two categories of variables: latent variables and observed variables. The latent variables were self-reported oral health, social support, and life satisfaction. The three observed variables for self-reported oral health included physical functioning, psychosocial functioning, and pain and discomfort. The three observed variables for social support included subjective support, objective support, and social support utilization. The five observed variables for life satisfaction were the five items of the SWLS. All SEM analyses were performed using Amos 22.0. The following model fit indices were used to judge the fit of the hypothesized model: chi-square (χ^2^), degrees of freedom (DF), root mean square error of approximation (RMSEA), the goodness-of-fit index (GFI), the adjusted goodness-of-fit index (AGFI), the incremental fit index (IFI), and the comparative fit index (CFI).

## 3. Results

### 3.1. Sample characteristics

Table 1 presents the participants' sociodemographic traits. As outlined in Table 1, the MEFC surveyed were predominantly women (73.1%), with a high proportion in the 60–69 age group (79.3%). In terms of marital status, 87.9% had spouses. Most (85.6%) were from rural areas. The main reason for their migration was to care for their grandchildren (86.9%). We noted statistical differences by gender, age, education level, the reason for migrating, and *hukou*.

**Table 1:** Sociodemographic characteristics and urban-rural differences in sociodemographic characteristics, self-reported oral health, social support, and life satisfaction.

**Variable**	**Total**		**Rural**		**Urban**		**χ^2^/t**	***P***
	***N*** **(%)**	**M(SD)**	***N*** **(%)**	**M(SD)**	***N*** **(%)**	**M(SD)**		
	613 (100)		525 (85.6)		88 (14.4)			
**Gender**							17.950^a^	0.001
Male	165 (26.9)		125 (23.8)		40 (45.5)			
Female	448 (73.1)		400 (76.2)		48 (54.4)			
**Age (year)**							8.856^a^	0.012
60*-*69	486 (79.3)		424 (80.8)		62 (70.5)			
70*-*79	103 (16.8)		85 (16.2)		18 (20.5)			
80+	24 (3.9)		16 (3.0)		8 (9.1)			
**Marriage status**							0.860^a^	0.354
Married	539 (87.9)		459 (87.4)		80 (90.9)			
No spouse	74 (12.1)		66 (12.6)		8 (9.1)			
**Education level**							36.513^a^	0.001
Primary school and below	267 (43.6)		203 (38.7)		64 (72.7)			
Middle school	185 (30.2)		169 (32.2)		16 (18.2)			
High school and above	161 (26.3)		153 (29.1)		8 (9.1)			
**Migrant Time (year)**							1.006^a^	0.316
< 4.5	302 (49.3)		263 (50.1)		39 (44.3)			
4.5≥	311 (50.7)		262 (49.9)		49 (55.7)			
**Migrant reason**							18.315^a^	0.001
Taking care of grandchildren	533 (86.9)		469 (89.3)		64 (72.7)			
Non-taking care of grandchildren	80 (13.1)		56 (10.7)		24 (27.3)			
**Self-reported oral health**		54.95 (6.469)						
Physical function				17.21 (3.489)		18.18 (3.027)	−2.730^b^	0.007
Psychosocial function				24.03 (2.151)		24.50 (1.348)	−2.725^b^	0.007
Pain and Discomfort				13.41 (2.143)		14.03 (1.860)	−2.841^b^	0.005
**Social support**		38.89 (6.629)						
Subjective support				23.45 (4.757)		23.58 (5.003)	−2.210^b^	0.027
Objective support				8.44 (1.611)		8.7 (1.766)	−0.229^b^	0.819
Utilization of Social Support				6.86 (2.229)		7.43 (2.372)	−1.425^b^	0.155
Life satisfaction		27.87 (5.584)		27.74 (5.751)		28.67 (4.401)	−1.758^b^	0.081

^a^χ^2^ test, ^b^t-test; M, mean; SD, standard deviation; 4.5 years = the median of migrant time.

The mean scores for self-reported oral health, social support, and life satisfaction are shown in Table 1. The mean values of GOHAI score, social support, and life satisfaction score were 54.95 ± 6.649, 38.89 ± 6.629, and 27.87 ± 5.584, respectively. Moreover, we observed statistically significant differences between life satisfaction, the three sub-dimensions of self-reported oral health (physical functioning, psychosocial functioning, and pain and discomfort), the subjective social support dimension, and *hukou*.

### 3.2. Correlation matrix analysis between social support, self-reported oral health, and life satisfaction of the MEFC

Table 2 presents the bivariate correlations between the observed variables and the three latent variables (self-reported oral health, social support, and life satisfaction) for the MEFC, indicating an almost positive correlation. Among them, the sub-item of the life satisfaction scale, “I have gotten [sic] the important things I want in life so far,” had a higher correlation with the subjective dimension of social support, and the correlation coefficient between them was 0.239 (*P* < 0.01).

**Table 2:** The correlation matrix analysis between social support, self-reported oral health, and life satisfaction.

	**1**	**2**	**3**	**4**	**5**	**6**	**7**	**8**	**9**	**10**	**11**
1	1										
2	0.793^**^	1									
3	0.839^**^	0.842^**^	1								
4	0.744^**^	0.740^**^	0.794^**^	1							
5	0.710^**^	0.706^**^	0.741^**^	0.843^**^	1						
6	0.179^**^	0.169^**^	0.175^**^	0.131^**^	0.126^**^	1					
7	0.209^**^	0.223^**^	0.223^**^	0.239^**^	0.207^**^	0.243^**^	1				
8	0.158^**^	0.167^**^	0.129^**^	0.105^**^	0.109^**^	0.138^**^	0.445^**^	1			
9	0.085^*^	0.110^**^	0.100^*^	0.105^**^	0.127^**^	0.031	0.171^**^	0.156^**^	1		
10	0.152^**^	0.150^**^	0.167^**^	0.188^**^	0.233^**^	−0.004	0.098^*^	0.090^*^	0.457^**^	1	
11	0.170^**^	0.170^**^	0.162^**^	0.155^**^	0.173^**^	0.005	0.122^**^	0.109^**^	0.676^**^	0.569^**^	1

^*^P < 0.05, ^**^P < 0.01^**^, 1 = Most aspects of my life are close to my ideal; 2 = I have good living conditions; 3 = I am satisfied with my life; 4 = I have gotten the important things I want in life so far; 5 = If I could go back and retrace my life's steps, there is almost nothing I would want to change; 6 = Utilization; 7 = Subjective; 8 = Objective; 9 = Physical Function; 10 = Psychosocial Function; 11 = Pain and Discomfort.

### 3.3. Mediating effect analysis

**Figure 2:**
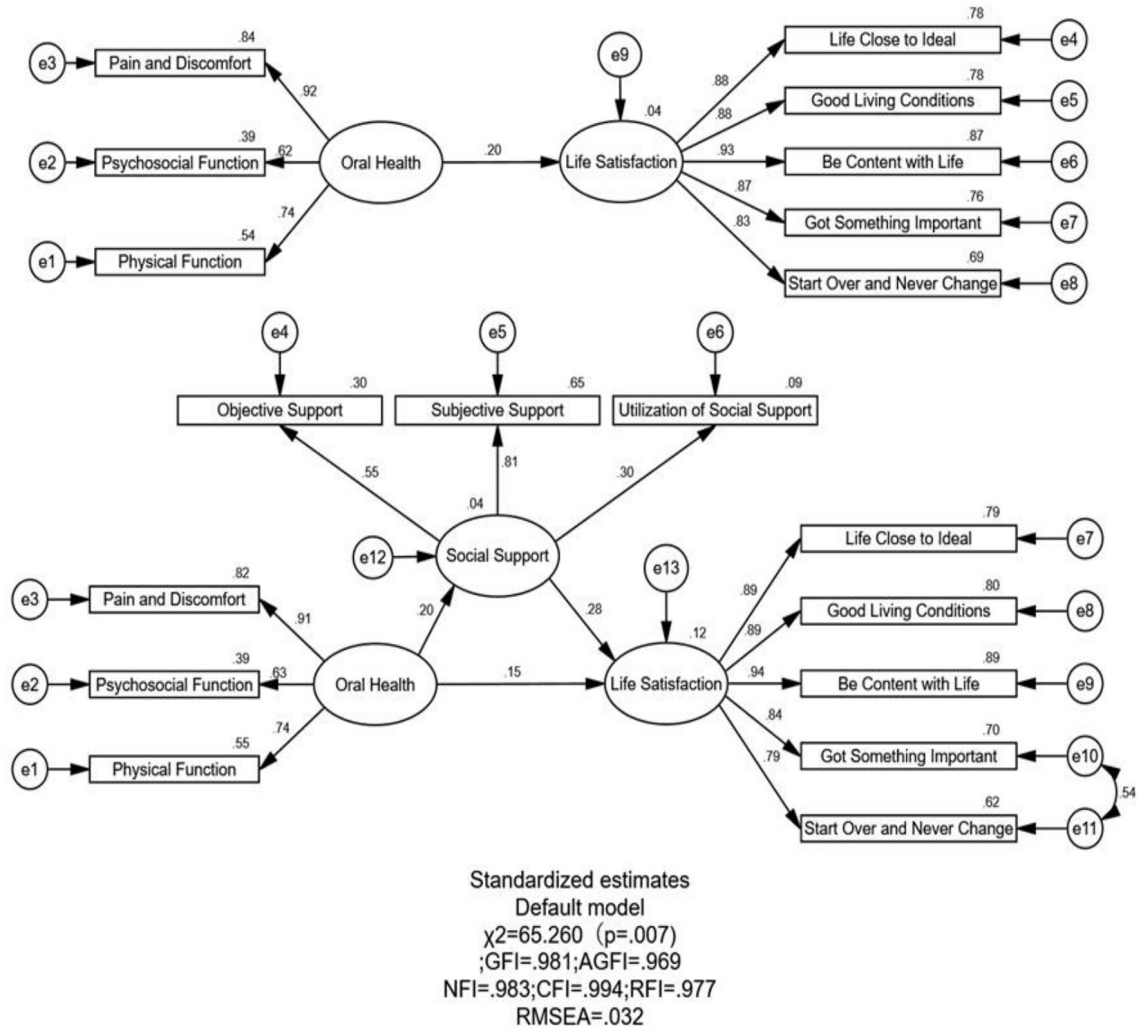
Path diagram of the association between oral health and life satisfaction of the MEFC in Weifang, China in with social support as a mediator. The coefficient in the parentheses are shown and all paths are statistically significant.

The model fit indices were checked before performing SEM analysis to ensure that the hypothesized model fitted the data well. As shown in Table 3 and Figure 2, χ^2^ = 65.260, *P* = 0.07, χ^2^/DF = 1.632, RMSEA = 0.032, CFI = 0.994, TLI = 0.991, all of which suggests that the model is a good fit.

**Table 3:** Fitting indexes of the model.

**χ^2^**	**DF**	**χ^2^/ DF**	**GFI**	**AGFI**	**IFI**	**CFI**	**RMSEA**	***P***
65.260	40	1-3	≥0.8	≥0.8	≥0.9	≥0.9	≤ 0.08	>0.05
		1.632	0.981	0.969	0.994	0.994	0.032	0.07

Table 4 and Figure 2 outlines the direct, indirect, and total effects on self-reported oral health and life satisfaction, between which there is a positive, direct relationship. This implies that the better the self-reported oral health of the MEFC, the greater their life satisfaction. There was also a positive, direct relationship between social support and life satisfaction, implying that the higher the social support of the MEFC, the greater their life satisfaction. In addition, there was a direct, positive effect on self-reported oral health and social support.

**Table 4:** The direct, indirect, and total effects of self-reported oral health and life satisfaction.

**Path**	**Standardized effect value**	**SD**	***P***	**Percent (%)**	**95%CI**	
					**LLCL**	**ULCL**
**Direct effect**	0.15	0.049	< 0.001	72.14	0.047	0.243
Self-reported oral health → life satisfaction						
**Indirect effect**	0.06	0.021	< 0.001	27.86	0.023	0.107
Self-reported oral health → social support						
Social support → life satisfaction						
**Total effect**	0.21	0.048	< 0.001	100	0.110	0.295

SD, standard deviation; CI, confidence interval; LLCL, lower limits confidence interval; ULCL, upper limits confidence interval.

Furthermore, the bootstrap tests showed that the total effect of self-reported oral health on life satisfaction among the MEFC was 0.21 (95% CI: 0.110–0.295, *P* < 0.001) after adjusting for covariates. The direct effect of self-reported oral health on life satisfaction among the MEFC was 0.15 (95% CI: 0.047–0.243, *P* < 0.001). The indirect mediating effect through social support was 0.06 (95% CI: 0.023–0.107, *P* < 0.001). These effects are significant because the 95% CI excluded zero. The association between self-reported oral health and life satisfaction is partially mediated by social support, and the mediating effect of social support accounts for 27.86% of the total effect.

## 4. Discussion

### 4.1. Principal findings

We aimed to examine the association between self-reported oral health and life satisfaction among the MEFC in Weifang, China, and to further explore the mediating role of social support between self-reported oral health and life satisfaction. Our results indicate that self-reported oral health not only influences life satisfaction among the MEFC directly but also indirectly through social support.

### 4.2. Self-reported oral health, social support, and life satisfaction of the MEFC

The mean GOHAI score was 54.95 ± 6.649. Specifically, 19.2% of MEFC patients had low self-reported oral health, 24.6% had moderate self-reported oral health, and 56.1% had high self-reported oral health. Hence, self-reported oral health still needs to be improved for a large proportion of the MEFC. One reason for low self-reported oral health may be aging, as previous studies have shown a negative correlation between aging and oral health (50). As humans age, oral health worsens (e.g., tooth loss). Another reason could be that most of the MEFC in this study were from rural areas (Table 1). Past research suggests that oral health is worse in rural areas, mainly in terms of lifestyle habits, and the MEFC may ignore oral hygiene issues and brush their teeth only once a day or not at all, leading to low self-reported oral health (51). Moreover, low self-reported oral health in rural areas may also be attributable to the availability and accessibility of oral health services or the lack thereof.

This study found that 76.3% of the MEFC (468 individuals) had a moderate level of social support, whereas 22.8% had an adequate level of social support. This outcome is similar to that of previous studies. A longitudinal study on social support for older adults found a decline in social support with increasing age (41). Most of the MEFC in this study may have had moderate levels of social support because their social networks shrank after they migrated to a new environment. They may have had fewer interactions with local residents due to language barriers and other issues and received less social support from their neighbors and friends (30).

The mean score of life satisfaction was 27.87 ± 5.584 in this study; this aligns with the result of a prior study on older adults in Tai'an City in Shandong Province, which found a life satisfaction score of 27.84 ± 5.32 (52). This may be because Weifang City and Tai'an City have similar economic development levels and have both been selected as the happiest cities in China (53). A study on Chinese empty nesters found that their life satisfaction score was 22.18 ± 5.87 (54), which implies somewhat higher life satisfaction among the MEFC in Weifang City. One possible reason is that the MEFC live with their children, whereas empty nesters live alone (55). A study on Korean immigrants who were older adults found that their life satisfaction score was 15.7 ± 4.49 (56), which was lower than that of the MEFC in this study. One reason may be that most of the MEFC came from rural areas, and their overall standard of living and medical services improved after migrating to big cities. Moreover, almost all the MEFC lived with their children, which helped in boosting their mental health (55).

### 4.3. The mediating role of social support in the relationship between self-reported oral health and life satisfaction of the MEFC

The SEM results indicate that self-reported oral health is associated with life satisfaction among the MEFC, which is consistent with a study by Ying et al. on Chinese patients with oral cancer (57). Self-reported oral health was found to be positively associated with life satisfaction among the MEFC (path coefficient = 0.15), which is similar to the outcomes of a study conducted among older adults in England (58). This suggests that higher self-reported oral health generally predicts greater life satisfaction among the MEFC. Thus, improving the self-reported oral health of the MEFC is beneficial for enhancing life satisfaction.

Our results suggest that self-reported oral health and social support are positively correlated. In other words, the higher the self-reported oral health of the MEFC, the higher their social support. This is in line with previous findings from a study of older adults in the United Kingdom, which found that social support is related to oral health status and health behaviors (59). This may be because of high self-reported oral health without tooth loss or mutilation, which reveals a better mental outlook and a greater willingness to communicate with others, thereby broadening one's social network.

The results also demonstrate a positive correlation between social support and life satisfaction (path coefficient = 0.28). This means that the higher the social support of the MEFC, the more likely they are to be satisfied with their lives. This finding aligns with the outcomes of a previous study on Korean immigrants (56). Another Korean study indicates that positive social relationships are enhanced through serious participation in activities, leading to increased life satisfaction (60). The results of a randomized trial conducted on older Japanese immigrants show that high social support is beneficial in reducing loneliness and boosting life satisfaction (61), which is consistent with our findings.

Finally, this study suggests that social support mediates the relationship between self-reported oral health and life satisfaction. This outcome is similar to the results of past studies. Findings from a study of older Korean immigrants imply that social support partially mediates the relationship between religiosity and life satisfaction and is an important predictor of life satisfaction (56). The results of a survey on older adults in Heilongjiang Province, China, reveal that social support fully mediates the effect of quality of life: the higher the level of social support received by older adults, the better their physical and psychological status and the greater their life satisfaction (62). Kooshiar et al. (63) also found that the social support function has a mediating effect on the relationship between living arrangements and life satisfaction among older adults in Malaysia (63). Another study has pointed out that the presence of emotional support may reduce the negative effects of disability on life satisfaction (64).

### 4.4. Implications

To enhance life satisfaction among the MEFC, the following steps should be taken. Firstly, self-reported oral health is an important component of quality of life that positively affects life satisfaction. As such, it is necessary to improve the self-reported oral health of MEFC patients. The government should speed up the process of including oral health services in health insurance reimbursements. Society can raise awareness of oral health among older adults through health education and publicity. Secondly, in this study, social support was found to positively correlate with life satisfaction and partially mediate self-reported oral health and life satisfaction, suggesting that social support is crucial to life satisfaction. Thus, there is a need to enhance social support for the MEFC from all parties, especially in the family. For example, children of the MEFC should not only receive financial support but also mental and emotional support. Thirdly, self-reported oral health and social support are directly and positively correlated. Therefore, for the MEFC, social support could be promoted by maintaining good oral hygiene, developing good oral habits, and having regular oral examinations.

### 4.5. Limitations

This study has several limitations. Firstly, the three scales used were subjective and lacked objective evidence. For instance, the clinical aspects of the oral health status of the MEFC were not examined. Therefore, there was no objective evidence upon which to make a judgment about the participants' oral health status. Secondly, a causal relationship between self-reported oral health, social support, and life satisfaction could not be established because this study was rooted in a cross-sectional design. Thirdly, social support partially mediates the relationship between self-reported oral health and life satisfaction, implying that other mediating variables may exist in this relationship. The potential mechanisms underlying the association between self-reported oral health and life satisfaction in older adults require further investigation. Fourthly, some information (such as the Hukou and jobs of the children of the MEFC and the family income) had not been included in this study; we will continue to add and improve upon such relevant information in future research. Lastly, due to the COVID-19 pandemic, the questionnaire survey was only conducted in Weifang and failed to begin in Shanghai as planned. In the future, the survey will be conducted in Shanghai if possible.

## 5. Conclusion

In summary, the mean score of life satisfaction was 27.87 ± 5.584 among the MEFC in Weifang, Shandong Province, China, which indicates that life satisfaction is relatively high among the MEFC. Our findings highlight an empirical association between self-reported oral health and life satisfaction and indicate that social support mediates this relationship. Our targeted recommendations can improve self-reported oral health, social support, and life satisfaction among the MEFC in China.

## Data availability statement

The datasets presented in this article are not readily available because our study was funded by National Natural Science Foundation of China, the application and usage of the data should be in accordance with the rules and requirements of the National Natural Science Foundation of China. Requests to access the datasets should be directed to FK, kongfanlei@sdu.edu.cn.

## Ethics statement

The studies involving human participants were reviewed and approved by the Institutional Review Board (IRB) of Public Health and Preventive Medicine in Shandong University. The patients/participants provided their written informed consent to participate in this study.

## Author contributions

JW analyzed the data, drafted the manuscript, and joined the data collection. MP joined the data collection, gave advice on statistical analysis, data processing, and comments on the modification of the manuscript. FK provided funding to support this study, designed the study, completed the questionnaire design, supervised and combined the data collected, instructed the writing, statistical analysis, data processing, and provided comments on the modification of the manuscript. All authors read and approved the final manuscript.
